# Assessment of the knowledge, attitude and practices of prescribers regarding malaria diagnosis: a cross sectional study among Ghanaian prescribers

**DOI:** 10.11604/pamj.2019.34.207.19940

**Published:** 2019-12-19

**Authors:** James Kojo Prah, Atta Yeboah-Sarpong, Richard Pinkrah, Elias Ewudzi-Acquah

**Affiliations:** 1 University of Cape Coast Hospital, Cape Coast, Ghana; 2 Ghana Ports and Harbors’ Hospital, Takoradi, Ghana

**Keywords:** mRDT, microscopy, knowledge, prescribers, Cape Coast

## Abstract

**Introduction:**

Malaria has proven to be the most fatal parasitic disease known to man. Among the pillars to malaria control are early and accurate diagnosis. In 2010, the World Health Organization launched its test, treat and track initiative which seeks to ensure that all suspected cases of malaria are tested. However, after several years of implementation, the use of malaria tests in diagnosing malaria has not been optimum. This study was conducted to assess the level of knowledge of prescribers on malaria Rapid Diagnostic Test and microscopy and to determine factors influencing prescribers' decision to request and use malaria tests in practice.

**Methods:**

A cross sectional study was carried out among 100 prescribers of various categories working in 4 hospitals in Ghana in March 2019. A pre-tested self-administered questionnaire was used to collect information on knowledge, malaria diagnostic practices and challenges faced by prescribers regarding parasitological testing for malaria in their health facilities.

**Results:**

Overall, 73% of respondents had good knowledge on malaria diagnostics. Routine use of malaria tests in diagnosing malaria was reported as 84%. Only 9% reported complete reliance on test results. Most participants (90%) reported awareness of the test-based case management of malaria.

**Conclusion:**

This study demonstrated that even though there was a high level of awareness of the test-before treatment policy among prescribers, significant numbers did not routinely request a malaria test for all suspected cases of malaria. Factors cited as barriers by prescribers were both health worker and health-system related that are all potentially modifiable.

## Introduction

Malaria continues to be the most dangerous parasitic disease afflicting man. According to the World Health Organization (WHO) in 2017, there was an estimated 219 cases of malaria worldwide with about 435000 deaths occurring as a result of malaria [1]. In Ghana, malaria is hyper-endemic and continuous to impose a major health and economic burden on the people. Malaria accounted for 38.1% of all out patient illness and 27.3% of all admissions in 2015 according to the National Malaria Control Programme (NMCP) annual report [2]. There is a regional variation in malaria parasite prevalence among children aged 6-59 months. The Eastern and Central regions have the highest (31%) and the second highest (30%) malaria prevalence, followed by the Volta (28%) and Northern (25%) regions. Malaria prevalence is lowest (5%) in the Greater Accra region [3]. Meanwhile, some researchers have discovered over-diagnosis and overtreatment of malaria as one of the main reasons for the higher cases [4-6]. Following evidence of over-diagnosis of malaria, the WHO launched the T3: Test. Treat. Track initiative. This global initiative was developed to urge all malaria endemic countries, donors and the global malaria community to scale up diagnostic testing, treatment and surveillance for malaria. Every suspected malaria case is therefore to be tested and every confirmed case should be treated with a quality antimalarial medicine [7]. In its annual report in 2010, the national malaria control programme (NMCP) of Ghana stated that presumptive diagnosis of malaria was very high and most febrile illness were wrongly captured as malaria. Ghana therefore adopted a test-based management of malaria and is expected that all prescribers confirm all suspected cases of malaria before commencing treatment for malaria [8].

Considering the fact that, the cost of using artemisinin-based therapies to treat malaria is very expensive, over-diagnosis of malaria coupled with its attendant over-treatment increases the economic burden of the disease. There is also a concern for possible resistance of the Plasmodium parasites to the artemisinin-based combination therapies. The NMCP reports that Ghana has recorded a rise in the proportion of OPD malaria cases tested by microscopy or RDT since 2012 from a low figure of 38.9% in 2012 to 73.6% in 2015. However, there is still more to be done so as to achieve the NMCP target of reducing malaria morbidity and mortality by 75% and to provide parasitological diagnosis to all suspected malaria cases and provide prompt and effective treatment to 100% of confirmed malaria cases by 2020 [2]. This objective cannot be achieved if prescribers continue to treat malaria without laboratory evidence. To achieve this objective, it is important that all prescribers involved in caring for patients with fever possess adequate knowledge of the various malaria diagnostic tests available in their facilities. Previous studies in Ghana have shown the reluctance of some clinicians to test before treating every suspected case of malaria. There is however paucity of information in all literature reviewed on the knowledge level of prescribers in Ghana on microscopy and mRDT as well as their attitudes and practices towards the use of these malaria diagnostic methods. This study was therefore conducted to determine factors that influence adherence of prescribers to the test-before-treatment policy of treating malaria in Ghana.

## Methods

**Study design:** this was a cross sectional study conducted in March 2019. The study made use of a semi-structured self-administered questionnaire.

**Population:** all prescribers working in four major hospitals in two different regions in Ghana were eligible for recruitment into the study. These hospitals are located in the capital cities of two regions (Central and Western) which all lie along the coast of Ghana. Malaria is hyper endemic in these areas. The selected hospitals have large numbers of prescribers and were therefore suitable for this cross-sectional study. The selected hospitals in the central region were the Cape Coast Teaching Hospital which is a tertiary level health facility with an estimated 60 prescribers and the University of Cape Coast Hospital which is a secondary level hospital with an estimated 15 prescribers. In the western region of Ghana, the selected hospitals were Efia Nkwanta Hospital which is the Regional hospital with an estimated number of 30 prescribers and the Ghana Ports and Harbors hospital with an estimated number of 5 prescribers.

**Sampling:** total population sampling technique was used. In this type of purposive sampling, the entire population of prescribers at all study sites were eligible for recruitment into the study. Prescribers working outside the selected hospitals and those not willing to participate were excluded.

**Study Instrument:** a semi-structured self-administered questionnaire developed from existing literature [9-11] was used. Steps were taken to validate the questionnaire in the population. These included establishing face validity by asking experts in malaria diagnostics to go through it. The questionnaire was then pre-tested using 10 prescribers at the Cape Coast Metropolitan hospital. Cronbach Alpha of 0.78 was found after test for the internal consistency of the questionnaire was carried out. The questionnaire was arranged into the following sections: (a) socio-demographic information; (b) knowledge on malaria rapid diagnostic tests and microscopy; (c) malaria diagnostic practices of prescribers; (d) training issues and challenges and (e) attitudes of prescribers towards malaria tests as well as recommendations of prescribers that will improve malaria diagnosis. The socio-demographic section was used to collect data such as age, sex, profession and years of practice of respondents. Prescribers' knowledge on malaria diagnostic tests was assessed using six questions. A correct answer scored 1 and a wrong answer scored 0. An “I don't know” response attracted a score of 0. A respondent was deemed to have adequate knowledge if he scored more than 50% (at least 4 out of 6).

**Ethical consideration:** ethical clearance was obtained from the Institutional Review Board of University of Cape Coast (UCCIRB). The main areas of concern in ethical involvement with participants included the issues of privacy, anonymity and confidentiality. These issues were addressed by training all those involved in the study to ensure confidentiality. Also, no names, staff numbers or any form of personal identification were used. A coding system was used to identify participants. The study was funded by the investigators. None of the investigators have declared any conflict of interest with regards to this study. Permission was sought from the management of the various hospitals before data collection began. Informed consent was obtained from each respondent before the administration of questionnaires.

**Data analysis:** data collected was entered into SPSS version 20. Descriptive statistics was used to summarize the data. Pearson's chi-square was used to examine the relationship between prescribers' demographic characteristics and their knowledge level on malaria diagnosis. Level of significance for all tests of association was put at p<0.5.

## Results

Out of a total of 110 questionnaires were distributed, 100 prescribers responded giving a response rate of 90.9%. Most of the participants were house officers (57.0%), followed by general practitioners (31.0%). Participants were aged between 24 years and 63 years with a mean age of 29.7± 6.2 years. Majority of respondents were in the age group of 26-30 years. Male prescribers were in the majority (63.0%). Clinical experience of participants ranged from <1 year to over 10 years. The socio-demographic characteristics of respondents are summarized in Table 1.

**Table 1:** Frequency distribution of socio-demographic characteristics of the respondents (N = 100)

Characteristic	Frequency	Percentage
**Age group (years)**		
20-25	14	14
26-30	60	60
31-35	15	15
36-40	6	6
41-45	2	2
>45	3	3
**Sex**		
Male	63	63
Female	37	37
**Health facility**		
Tertiary	60	60
Secondary	33	33
Primary	7	7
**Profession**		
Specialist	3	3
General practitioner	31	31
House officer	57	57
Physician assistant	6	6
Nurse practitioner	3	3
**Years of practice (years)**		
<1	36	36
1-5	49	49
6-10	6	6
>10	9	9

**Knowledge on malaria diagnosis:** the basic knowledge of participants on malaria diagnosis was assessed using 6 questions all carrying equal scores (Table 2). Participants obtained an average score of 4.06±1.03 out of a maximum score of 6. Modal score was 4 with a range of 1 to 6. A participant was deemed to have good knowledge if he scored more than 50%. Majority of the participants (73%) had good knowledge on malaria diagnosis. There was no significant difference in the scores obtained by the various categories of prescribers (Table 2). Most participants (93%) knew correctly that microscopy detect specifically the presence of malaria parasites in blood. Only 41% of respondents knew that mRDTs cannot be used to detect malaria if a certain threshold of parasitaemia is not met. When asked about the gold standard for malaria diagnosis in clinical practice, 84% knew correctly that it is microscopy. As many as 10 participants reported that they had not heard about the WHO recommendation that all suspected cases of malaria must be tested prior to initiating treatment (Table 2).

**Table 2:** Basic knowledge of malaria diagnostic tests among respondents

Question	Profession	Correct answer (%)	Chi-square
Microscopy is used to detect specifically the presence of parasites in blood	Specialist	3(100)	0.53
	General practitioner	31(100)	
	House officer	53(92.9)	
	Physician assistant	6(100)	
	Nurse practitioner	3(100)	
**Total**		93	
Malaria RDT detect specifically the presence of antigens in blood	Specialist	2(66.7)	0.59
	General practitioner	20 (64.5)	
	House officer	34(59.6)	
	Physician assistant	3(50)	
	Nurse practitioner	1(33.3)	
**Total**		60	
Malaria RDTs can be used to diagnose malaria irrespective of the number of malaria parasites in blood	Specialist	2(66.7)	0.001
	General practitioner	10(32.3)	
	House officer	29(50.9)	
	Physician assistant	0	
	Nurse practitioner	0	
**Total**		41	
A negative microscopy test virtually rules out malaria	Specialist	2(66.7)	0.13
	General practitioner	9(29.0)	
	House officer	22(38.6)	
	Physician assistant	0	
	Nurse practitioner	1(33.3)	
**Total**		34	
Microscopy is the regarded as the gold standard in clinical practice	Specialist	3(100)	0.67
	General practitioner	25(80.6)	
	House officer	48(84.2)	
	Physician assistant	5(83.3)	
	Nurse practitioner	3(100)	
**Total**		84	
Have you heard about the WHO recommendation of testing all suspected malaria cases before commencing treatment?	Specialist	3(100)	0.59
	General practitioner	28(90.3)	
	House officer	51(89.5)	
	Physician assistant	6(100)	
	Nurse practitioner	2(66.7)	
**Total**		90	

**Mean scores of knowledge of malaria diagnosis:** among the participants, specialists score the highest mean score of 5.10 for knowledge on diagnosis assessment. House Officers followed with a mean score of 4.10. General practitioners had a mean score of 3.97, whilst physician assistants and nurse practitioners had mean scores of 3.33 and 3.07 respectively. There was a significant relationship between knowledge on malaria testing and request for malaria test (x^2^=0.039).

**Malaria diagnostic practice:** when asked what the main method of malaria diagnosis in their health facility was, majority of participants (48%) mentioned the use of both mRDT and microscopy whilst 9% relied mainly on clinical judgment to diagnose malaria (Table 3). Most participants (84%) said they routinely test all cases of suspected malaria whilst 16% do not. With regards to factors considered by participants before requesting a malaria test, severity of patients' disease was mentioned by 77% of respondents followed by workload at the clinic (36%). Only 9% of participants said they always comply (100%) with malaria test results in managing their patients. In the presence of a negative test result 93% said they might still treat for malaria if they had a strong clinical suspicion, whilst 23% said they might still treat for malaria because they have doubts in the accuracy of test results. One participant reported he will treat for malaria if the patient preferred it even in the absence of a positive malaria test. Table 3 summarizes responses of respondents to questions pertaining to malaria diagnostic practices.

**Table 3:** Responses of participants to questions on malaria diagnostic practices

Question	Responses	Frequency (%)
Which of the following methods are mostly used in diagnosing malaria in your facility	Clinical	9
	mRDT	17
	Microscopy	15
	Both mRDT and microscopy	48
	Clinical, mRDT and microscopy	11
Do you routinely request for malaria test when you suspect uncomplicated malaria in your patients?	Yes	84
	No	16
What factors do you consider before requesting a malaria lab test?	Severity of disease	77
	Work load at the clinic	36
	Age of patient	20
	Availability of laboratory services	15
How often do you rely completely on your test results in treating your patients?	100%	9
	80-99%	60
	50-79%	29
	<50%	2
What factors may cause you to treat malaria in the presence of a negative test result?	Strong clinical suspicion	93
	Test result may be inaccurate	23
	Age of patients	11
	Patient’s preference	1

**Training issues and challenges:** only 17% of participants said they had been trained on the WHO T3 strategy. There was no significant association (x2=0.068) between years of practice and being trained on the WHO T3 strategy. Majority of participants (70%) said they had not ever received any training on mRDT and microscopy. Most participants (89%) could however perform a malaria RDT test confidently without supervision, perhaps learning the skill from others at work. On challenges faced by prescribers in their use of laboratory investigations to diagnose malaria, unavailability of mRDT all the time was most cited (59%), followed by mistrust in test results of mRDT (48%) and microscopy (27%). Regarding attitudes of prescribers towards malaria lab tests, most (61%) viewed the tests as very helpful whilst 37% thought it is sometimes helpful. A minority of 2% said it was not helpful. On their view of the WHO policy of testing all suspected cases of malaria before treatment, majority of the prescribers surveyed said it was a very good policy whilst 25% thought it restricts clinical skills. Table 4 shows responses to questions pertaining to training on malaria tests and challenges faced by prescribers. Recommendations of participants to improve malaria diagnosis in Ghana have been summarized in Table 5. Most (60%) of participants mentioned that RDT kits must be readily available at all levels of care in the country, followed by adequate training for all staff especially laboratory technicians and prescribers (50%).

**Table 4:** Responses to questions pertaining to training on malaria tests and challenges faced by prescribers

Question	Response	Frequency
Have you ever been trained on the WHO T3 strategy?	Yes	12
	No	88
Have you received training on the use of RDTs and microscopy for testing for malaria?	Yes	30
	No	70
Can you confidently perform mRDT for a patient without assistance	Yes	89
	No	11
Are there any challenges you have faced with the use of lab tests in diagnosing malaria in your facility?	mRDTs are not always available	59
	Blood film results cannot always be trusted	27
	RDTs not giving accurate results	48
	Lack of trained staff	5

**Table 5:** Participants’ recommendations on malaria diagnosis

Recommendation	Frequency (%)
All health facilities should have RDTs readily available always	60
There should be adequate training for laboratory technicians on microscopy	50
There should be more training for prescribers	45
Waiting time for microscopy results should be reduced	38
RDTs that are able to detect more species should be used	36
Accuracy of RDTs should be increased	30
There should be more education on malaria testing for the general public	22

## Discussion

The primary aim of this study was to determine the knowledge level of prescribers on malaria diagnosis. The study revealed a high level (73%) of adequate knowledge on malaria diagnosis among participants. This is higher compared to earlier studies conducted among prescribers in Zamfara State [12] and Enugu State [13] in Nigeria. These studies found mean scores of 64.7% and 70.0% respectively. The study at Zamfara State was conducted among various categories of health workers including laboratory scientists/technicians, community health extension workers/community health officers, nurses and doctors whilst the Enugu State study focused only on medical doctors. The current study involved doctors, physician assistants and nurse practitioners. The previous studies aimed at assessing knowledge on mRDT alone whilst this study determined knowledge on both microscopy and mRDT. The differences in mean scores obtained in the various studies cited could be due to the different questions used in the assessment of knowledge and different scoring criteria used. Good knowledge of malaria diagnostics has been found to directly influence positively test utilization among care givers [12]. It is significant to note that almost all prescribers indicated that they were aware of WHO's recommendation of testing all suspected malaria cases before treatment. This could probably be due to the intense education mounted by the Ghana National Malaria Control Programme (NMCP) especially in the media. In its first quarter of 2017 report, the NMCP reported that it used mass media campaigns to advocate and intensify education on the test, treat and track strategy so as to increase compliance, use and improve provider confidence in malaria tests. There was a total of 54 television and 1817 radio adverts aired across the country [14].

However, the study found that not all prescribers routinely request parasitological tests for all suspected cases of malaria before commencing treatment. This finding agrees with what were found in some previous studies in Ghana [9,15] and Papua New Guinea [16] that reported the reluctance of some prescribers to comply with national guidelines for malaria case management that includes testing all suspected malaria cases before treatment. Most respondents in this study self-reported that they do not always relied on test results even if they requested them. This implies that they sometimes prescribe antimalarials to patients with negative test results. Other studies conducted across the globe have also made similar findings. An earlier study conducted in Ghana found a low prescriber compliance with the malaria test-based case management policy of Ghana [9]. In a Papua New Guinea study [16], 15.3% of febrile patients who presented negative malaria test results were treated for malaria. A study conducted in Nigeria by Uzochukwu *et al*. (2011), found that antimalarials were prescribed in 74% of RDT-negative results [17]. This study identified several barriers to the test-based management of malaria as reported by prescribers. These includes reliance of strong clinical suspicion of malaria in patients, mistrust in parasitological tests and increasing workload at clinics. It is estimated that on the average, 24885 suspected cases of malaria report to health facilities in Ghana. Testing all these suspected cases will no doubt increase workloads to prescribers as stated by participants in this study.

Most respondents had not received any formal training on the use of mRDTs and microscopy. These findings have been confirmed among clinicians in other parts of Ghana [9,15]. A Sudanese study revealed that 76% of providers in Sudan had not received formal training on the use of mRDT [6]. There is therefore a need for a multifaceted approach to deal with this issue of prescriber non-compliance to national guidelines. The approach should involve the main stakeholders in malaria care who are prescribers, management of health facilities, government and non-governmental organizations. The National Malaria Control Programme (NMCP) of Ghana should increase training of clinicians in the country on malaria diagnosis and the WHO T3 strategy as a whole. The training should be aimed at addressing the concerns of prescribers that have served as barriers to their compliance to the national guidelines. The finding of only 12% of respondents having had formal training on the WHO T3 strategy is alarming and must be addressed with alacrity. The NMCP in their 2016 Annual Bulletin indicated that it conducted case management training, Clinical Outreach Training and Supportive Supervision (OTSS) in five Regions (Brong Ahafo, Greater Accra, Upper East, Upper West, Volta and Western Regions) in Ghana with a total of 12,159 health staff trained. Trainer of Trainers (TOT) was also conducted for eighteen Regional coordinators of Ghana Registered Midwives Association (GRMA) [2]. Case management refresher trainings were carried out for 5,781 health workers in Greater Accra, Northern, Volta and Western Regions in collaboration with Systems for Health, a USAID/PMI supported project [2]. Even though these trainings are very commendable, it is clear from the findings from this study that more such trainings are needed especially for prescribers. Some prescribers in this study reported they cannot perform RDTs confidently on their own without supervision. It has been documented in earlier studies [18,19] that training of health workers on mRDT improves their likelihood of adherence to malaria treatment guidelines.

Most prescribers mentioned the sporadic and inadequate availability of RDTs as a major challenge in their adherence to the guidelines. This challenge has been cited as a major barrier to the compliance to national guidelines by clinicians in many previous studies conducted in other malaria endemic low-income settings [20,21]. Unavailability mRDT and microscopy services will in no doubt encourage prescribers to resort to presumptive treatment of malaria. It is widely documented that a very important factor in improving malaria diagnosis is the availability of malaria diagnostic tests in health facilities [22]. In Ghana RDTs are provided by the government and some NGOs for free to Public health facilities. The government of Ghana and its partners through the NMCP must therefore address the shortage of the RDT kits and scale-up mRDTs availability to private facilities as well. Among respondents found to have inadequate knowledge on malaria diagnosis were newly trained doctors who are expected to be up to date with current management strategies. This finding means that training institutions for doctors, physician assistants and nurse practitioners must give more attention to training on malaria given the public health significance of malaria in our setting. Studies have shown that young clinicians are more likely to comply with current case management guidelines compared with older colleagues [23]. Thus, the best strategy to achieve a behaviour change with regards to malaria tests is to inculcate the policy in clinicians whilst still undergoing training at school. There was a general consensus among the respondents in this study that the WHO policy on test-based management of malaria is good. This is an endorsement suggests that when steps are taken to address the barriers identified in this study the aim of the NMCP of provision of parasitological diagnosis to all suspected malaria cases and prompt effective treatment to 100% of confirmed malaria cases by 2020 can be achieved.

**Limitations of study:** the study made use of a questionnaire which was used to capture self-reported experiences, hence primarily relied on provision of the right information by respondents. There is a possibility of reporting bias as respondents may provide socially desirable answers. This was however minimized by assuring a high degree of confidentiality. Also, the study did not verify information provided by participants as case notes were not examined and also patients were not interviewed. Further studies need to be conducted in our setting aimed at assessing the availability of mRDTs and microscopy services. Such studies should include direct observation of clinicians at work and a follow up review of patients' notes to ascertain if malaria tests were requested and if the results were used in the management of the patients.

## Conclusion

There was a high level of adequate knowledge and awareness on malaria diagnosis among participants. However, there were some significant numbers of prescribers who did not routinely request for malaria tests for all suspected cases of malaria. This suggest that their non-compliance to the test before treatment policy could probably not be attributed to lack of awareness and knowledge but rather is attitudinal and therefore mitigating policies should be aimed at causing attitudinal change. Most prescribers in this study admitted that they do not always rely on malaria test results. This implies that they sometimes prescribe anti-malarial drugs in the presence of negative malaria test results. There was a high proportion of clinicians who have never received any formal training on the WHO T3 strategy. This is worrying as it stands to potentially derail all efforts targeted at defeating malaria in Ghana.

### What is known about this topic

- Test-based management of malaria is practiced in Ghana;
- There is over diagnosis of malaria among prescribers in Ghana.

### What this study adds

- Prescribers have adequate knowledge on methods of diagnosing malaria;
- Some prescribers do not routinely request malaria test for all suspected cases of malaria;
- This study identified several barriers to the test-based management of malaria as reported by prescribers.

## Competing interests

The authors declare no competing interests.
